# Mr. Custance, on the Hæmorrhoids

**Published:** 1800-04

**Authors:** Geo. Custance

**Affiliations:** Kidderminster


### *To the Editors of the Medical and Physical Journal.*

Gentlemen,

SHOULD the following observations be thought worthy a place in your useful Journal, you will oblige me by inserting them in your next Number. I am,

Gentlemen,

Your's respectfully,

GEO. CUSTANCE.

*Kidderminster,*

*March* 8, 1800.

---

PERHAPS there is no disease incident to the human body, more general, or more troublesome, than the Piles. Not only are aged persons and pregnant women often tormented by this painful disorder, but young people are very frequently subject to it. It is peculiarly distressing to many young females, who, from a sense of delicacy, conceal their disorder, or make it known to none but some female friend, who temporizes with the trial of a variety of nostrums which afford little or no relief.

I know of no disorder, in the treatment of which medical men appear more implicitly to have imitated their predecessors.

Lenitive

Lenitive electuary, lac fulph. and nitre, have been the reigning *triumvirate* fo long, that any attempt to dethrone them may be deemed high treafon againft their eftablifhed authority. I allow, that when Piles are the mere effect of pregnancy, thefe aperient medicines may be very ufeful, and all that is needful; but experience proves, that purgatives afford no further relief than removing occafional coftivenefs, which, I am of opinion, is more frequently the effect than the caufe of Piles in men, and in women not pregnant. Dr. Cullen, and others, have confidered the Piles as depending on *conftitutional* plethora; but, with deference to fuch high authority, I am inclined to think they are a *local* affection of the rectum. Dr. H. Smith has defined the Piles to be "a difeafe which derives its origin from an effufion of blood into the cellular membrane of, and furrounding the rectum;" and advifed "anodyne and repellent *liniments* and *fomentations*, keeping open the body by gentle, cooling purgatives;" thefe often prove palliatives where there are any external excrefcences, but will generally be found ineffectual for the *inward* Piles. Confidering the fulnefs and pain which are felt in this ftate of the diforder, to depend on a relaxed ftate of the coats of the rectum, occafioning a flower circulation of the blood, in that part, than in the healthy ftate, I have long been in the habit of adminiftering anodyne and aftringent *injections*, which I have found very fuccefsful in completely removing the Piles, efpecially in young perfons. The injection I always ufe is the following; the proportion of each ingredient being varied according to circumftances:

℞. Tinct. Ferri muriati ʒij; tinct. opii ʒi to ʒij; decoct. hordei ℥iv. M. ft. enema bis de die injiciend.

Proper attention being paid to diet will commonly prevent coftivenefs; and bark, with other ftimulants, I am perfuaded, will be found more beneficial than purging medicines; as, by ftrengthening the tone of the vifcera, their natural functions are gradually reftored. It is manifeft that thefe injections are not propofed as remedies for external excrefcences, which may be removed by ligature, in moft cafes, without any danger.

